# Supplementary material for: Tissue context determines the penetrance of regulatory DNA variation
Source: Nat Commun. 2021 May 14;12:2850. doi: 10.1038/s41467-021-23139-3 (PMC8121920; doi:10.1038/s41467-021-23139-3)
Supplement: Supplementary file 1 — Supplementary Information [file 41467_2021_23139_MOESM1_ESM.pdf]

Supplemental material for:

Tissue context determines the penetrance of regulatory DNA variation

TABLE OF CONTENTS

SUPPLEMENTAL FIGURES **2**
Supplementary Fig. 1. Fragment length distributions of DNase-seq data 2
Supplementary Fig. 2. DNase-seq data replicate concordance. 3
Supplementary Fig. 3. Summary of allelic imbalance analysis. 4
Supplementary Fig. 4. Imbalance versus read depth and number of samples. 5
Supplementary Fig. 5. Rates of imbalance for various genomic features. 6
Supplementary Fig. 6. CATO2 performance on mESC validation data. 7
Supplementary Fig. 7. CATO2 predictive performance for variation affecting TF occupancy 8

SUPPLEMENTARY TABLES **9**
Supplementary Table 1. Summary of DNase I samples in this study. 9
Supplementary Table 2. Summary of DNase I data by strain and tissue type. 10
Supplementary Table 3. Summary of TF models. 10
Supplementary Table 4. Summary of mESC samples in this study. 11
Supplementary Table 5. Summary of RNA-seq samples in this study. 11
Supplementary Table 6. PCR primers. 11

SUPPLEMENTAL FIGURES

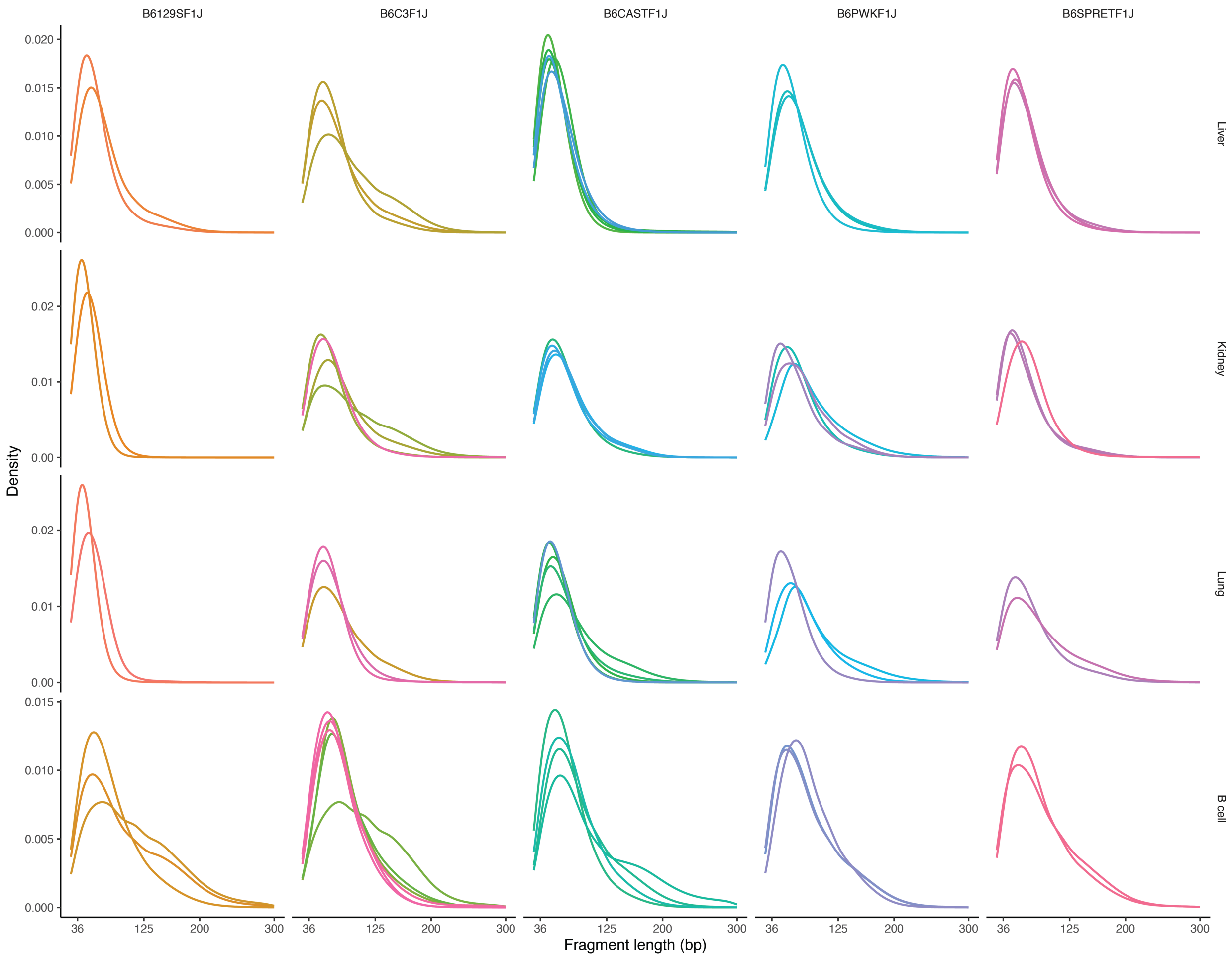

Supplementary Fig. 1. Fragment length distributions of DNase-seq data.

Shown are samples passing all QC filters.

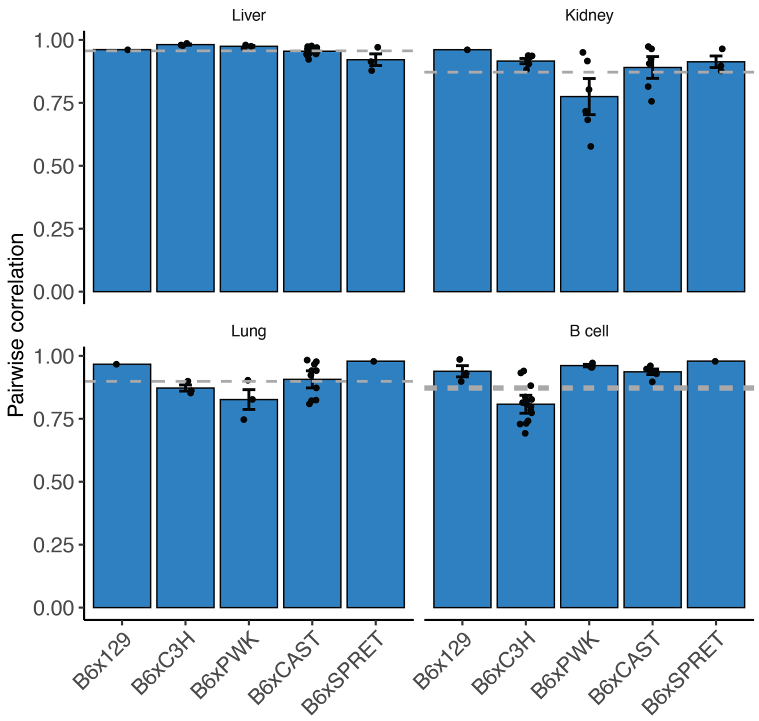

Supplementary Fig. 2. DNase-seq data replicate concordance.

Pairwise replicate concordance for each cell/tissue type and strain. Y-axis measures the pairwise Pearson correlation between pairs replicates of DNase cleavage density in hotspots. Bars summarize all replicate pairs and are presented as mean ± standard deviation.

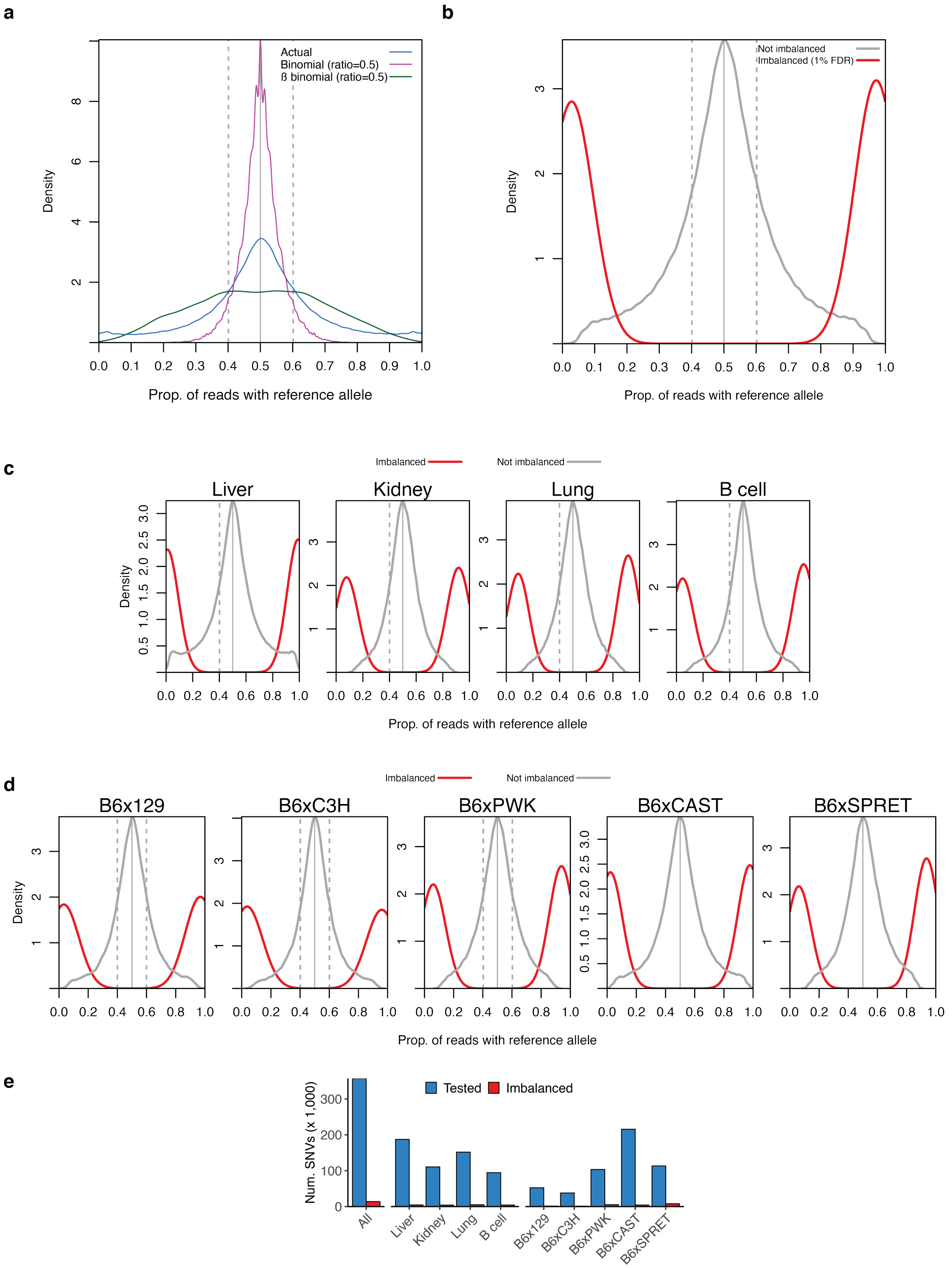

Supplementary Fig. 3. Summary of allelic imbalance analysis.

a. Distribution of allelic ratio for actual data compared to data simulated from binomial and beta-binomial distributions. **b**-**d**. Distribution of allelic ratios for aggregate (**b**), per-cell type (**c**), per-strain (**d**) analyses. **e**. Counts of SNVs tested for imbalance (blue) and significantly imbalanced SNVs (red, FDR 10%). Counts are reported in aggregate across all data sets (left), by cell type (middle), and by parental strain (right).

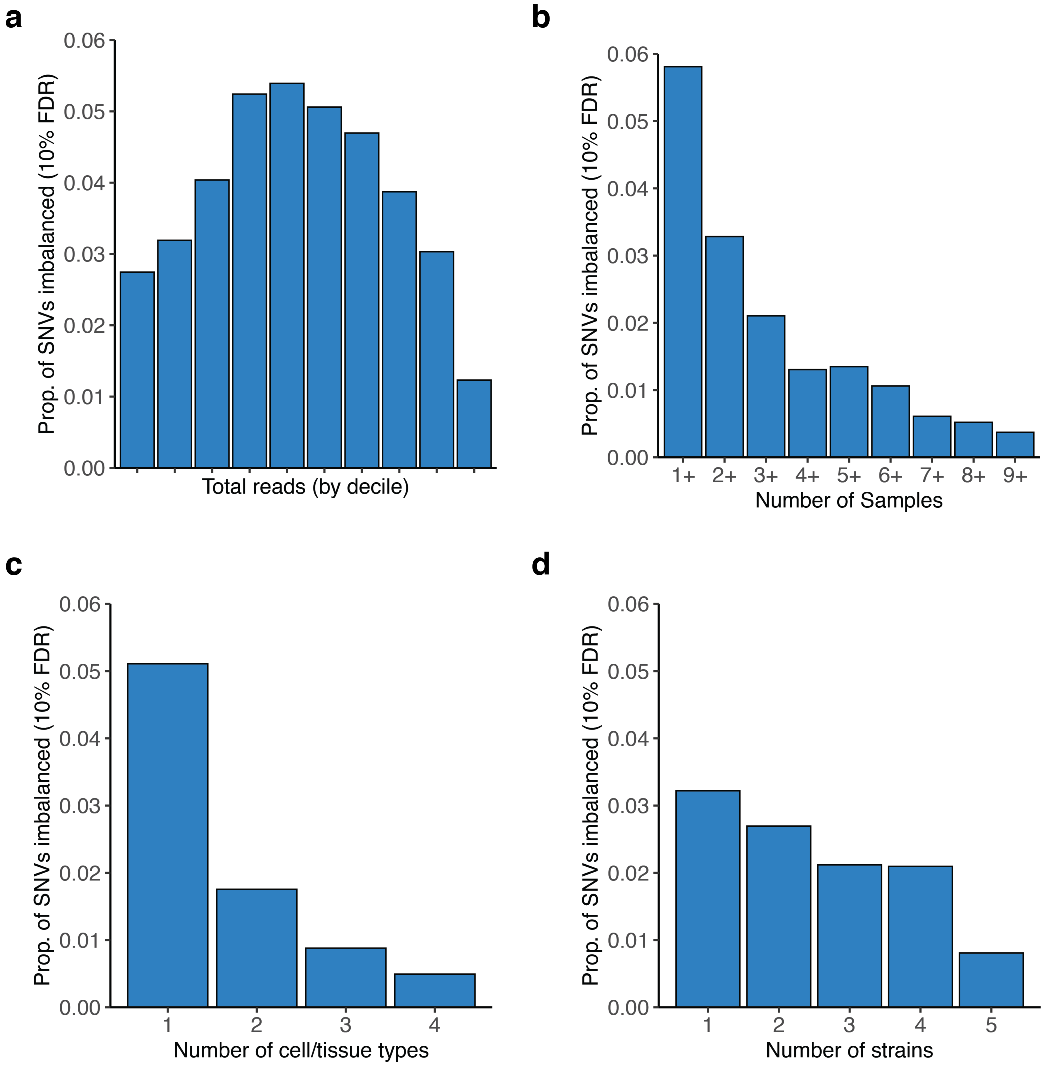

Supplementary Fig. 4. Imbalance versus read depth and number of samples.

a. Frequency of imbalance by total reads for that SNV across all samples. **b-d**. Frequency of imbalance by the number of samples (**b**), cell/tissue types (**c**), or strains (**d**) in which a SNV was measured. Data are summarized per Cell type (**a** and **c**, n=357,303 SNVs), by sample (**b**, n=304,360 SNVs), or by strain (**d**, n=404,215 SNVs).

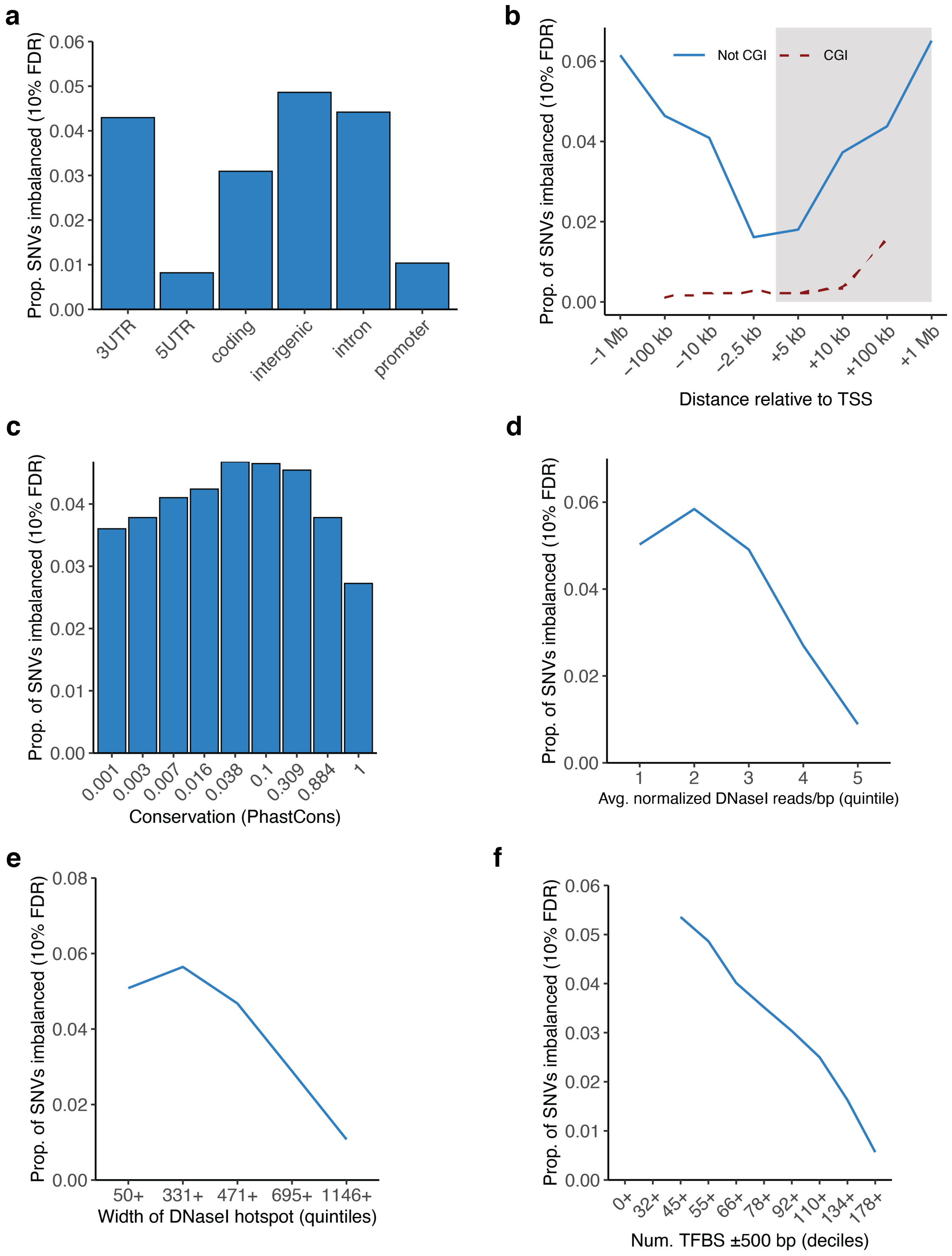

Supplementary Fig. 5. Rates of imbalance for various genomic features.

Frequency of imbalance relative to **a**. genic sequence, **b**. distance to transcription start site (TSS), **c**. phylogenetic conservation (PhastCons), **d**. DHS strength, **e**. DHS hotspot width, and **f**. number of nearby TFBS in footprints.

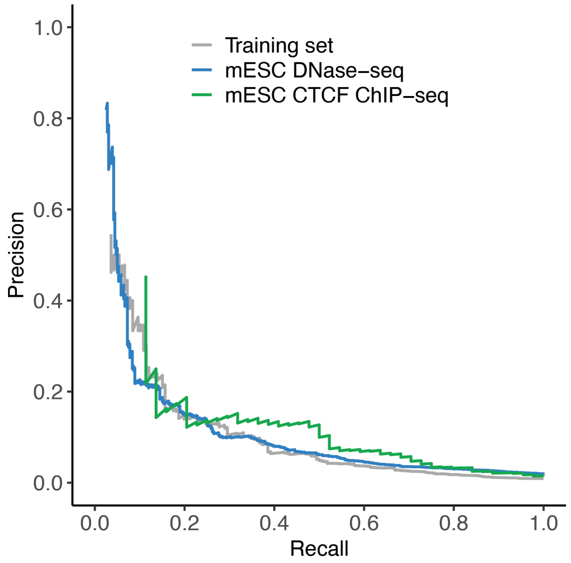

Supplementary Fig. 6. CATO2 performance on mESC validation data.

Precision-recall assessment of CATO2 predictions using independently generated allelic accessibility and CTCF occupancy from mESCs (n=18,211 and 2,995 SNVs tested for imbalance, respectively). Training set performance is shown at DHSs in common with mESC data. For ChIP-seq, only predictions overlapping CTCF recognition sequences (FIMO $P < 10^{-4}$) were assessed.

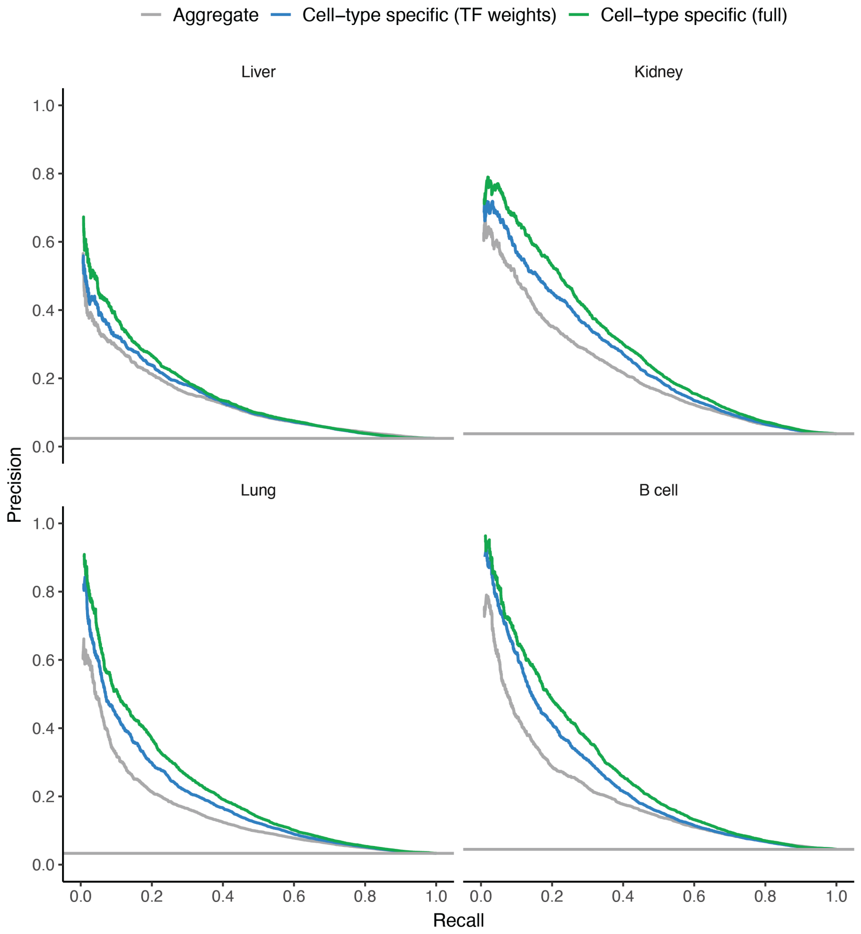

Supplementary Fig. 7. CATO2 predictive performance for variation affecting TF occupancy.

Precision-recall curves showing performance to predict imbalanced polymorphism. Shown are SNVs tested for imbalance in individual cell types. Solid lines represent performance of models trained using glmnet package to have cell-type specific weights for relevant TFs. Gray lines represent performance of a random classifier based on the proportion of true positives in dataset.

Supplementary Tables

Supplementary Table 1. Summary of DNase I samples in this study.

Analyzed reads were required to pass all mapping filters. Read counts are in millions. Signal Portion of Tags (SPOT) scores are a measure of enrichment and refer to the proportion of reads mapping within DHS; SPOT scores are reported from hotspot V1.

Strain	Cell/tissue type	Sample ID	# Sequenced reads (M)	# Analyzed reads (M)	# Duplicate reads (M)	SPOT
B6x129	B cell	DS33334	175	99	9	0.51
B6x129	B cell	DS33340	395	215	32	0.40
B6x129	B cell	DS33342	50	32	2	0.38
B6x129	Kidney	DS32758	176	121	49	0.76
B6x129	Kidney	DS32759	104	50	6	0.77
B6x129	Liver	DS32752	444	239	49	0.70
B6x129	Liver	DS32753	656	378	79	0.67
B6x129	Lung	DS32746	165	93	13	0.61
B6x129	Lung	DS32747	404	205	127	0.66
B6xC3H	B cell	DS34563	10	2	3	0.81
B6xC3H	B cell	DS34565	70	31	3	0.48
B6xC3H	B cell	DS34569	9	2	3	0.76
B6xC3H	B cell	DS38898	105	44	18	0.31
B6xC3H	B cell	DS38899	126	53	19	0.36
B6xC3H	B cell	DS38900	151	58	25	0.45
B6xC3H	Kidney	DS33566	211	105	17	0.63
B6xC3H	Kidney	DS33567	34	7	0	0.81
B6xC3H	Kidney	DS33568	154	95	6	0.46
B6xC3H	Kidney	DS38819	33	12	4	0.81
B6xC3H	Liver	DS33560	247	98	7	0.71
B6xC3H	Liver	DS33561	488	278	98	0.70
B6xC3H	Liver	DS33563	65	33	1	0.59
B6xC3H	Lung	DS33554	178	68	10	0.36
B6xC3H	Lung	DS38811	131	63	49	0.69
B6xC3H	Lung	DS38812	152	66	44	0.50
B6xCAST	B cell	DS35978	459	194	93	0.79
B6xCAST	B cell	DS35986	41	22	4	0.58
B6xCAST	B cell	DS35992	63	27	6	0.74
B6xCAST	B cell	DS35993	303	139	66	0.71
B6xCAST	Kidney	DS35927	75	25	2	0.49
B6xCAST	Kidney	DS36776	279	129	10	0.48
B6xCAST	Kidney	DS36777	138	65	4	0.49
B6xCAST	Kidney	DS36778	378	186	18	0.46
B6xCAST	Liver	DS35877	278	154	28	0.69
B6xCAST	Liver	DS35884	137	77	10	0.57
B6xCAST	Liver	DS35889	77	30	3	0.86
B6xCAST	Liver	DS35890	230	112	16	0.74
B6xCAST	Liver	DS36784	15	5	0	0.76
B6xCAST	Liver	DS36791	82	34	3	0.61
B6xCAST	Lung	DS35897	84	44	3	0.48
B6xCAST	Lung	DS35898	705	377	80	0.59
B6xCAST	Lung	DS35909	259	93	15	0.45
B6xCAST	Lung	DS35910	62	20	12	0.55
B6xCAST	Lung	DS36795	24	7	0	0.44
B6xPWK	B cell	DS36869	358	159	77	0.53
B6xPWK	B cell	DS36870	24	11	2	0.46
B6xPWK	B cell	DS36871	407	198	120	0.59
B6xPWK	Kidney	DS36635	279	147	34	0.60
B6xPWK	Kidney	DS36649	80	37	9	0.54
B6xPWK	Kidney	DS37495	73	13	2	0.66
B6xPWK	Kidney	DS37496	337	135	14	0.38
B6xPWK	Liver	DS36636	64	32	2	0.56
B6xPWK	Liver	DS36641	173	95	12	0.65
B6xPWK	Liver	DS36648	507	218	54	0.86
B6xPWK	Lung	DS36655	120	47	29	0.68
B6xPWK	Lung	DS36657	527	202	77	0.45
B6xPWK	Lung	DS37487	383	65	11	0.57
B6xSPRET	B cell	DS39204	176	58	8	0.55
B6xSPRET	B cell	DS39205	220	115	75	0.58
B6xSPRET	Kidney	DS37590	225	58	4	0.65
B6xSPRET	Kidney	DS37591	205	64	6	0.74
B6xSPRET	Kidney	DS39287	12	6	0	0.56
B6xSPRET	Liver	DS37603	292	139	15	0.61
B6xSPRET	Liver	DS38318	66	29	10	0.81
B6xSPRET	Liver	DS38327	178	71	36	0.83
B6xSPRET	Lung	DS37582	246	66	5	0.48
B6xSPRET	Lung	DS38311	197	104	7	0.38

Supplementary Table 2. Summary of DNase I data by strain and tissue type.

Strain	Cell/tissue type	Analyzed reads	# Biological replicates	# Hotspots (5% FDR)
B6x129	B cell	347,204,832	3	117,910
B6x129	Kidney	170,705,656	2	238,917
B6x129	Liver	617,160,439	2	295,565
B6x129	Lung	298,565,608	2	261,732
B6xC3H	B cell	190,135,121	6	78,387
B6xC3H	Kidney	218,833,896	4	203,372
B6xC3H	Liver	409,883,230	3	215,510
B6xC3H	Lung	198,062,728	3	152,006
B6xCAST	B cell	394,467,062	4	156,078
B6xCAST	Kidney	404,999,564	4	228,944
B6xCAST	Liver	413,748,454	6	233,784
B6xCAST	Lung	543,296,869	5	281,978
B6xPWK	B cell	376,482,554	3	134,316
B6xPWK	Kidney	332,674,371	4	231,416
B6xPWK	Liver	345,057,236	3	242,731
B6xPWK	Lung	315,239,886	3	194,034
B6xSPRET	B cell	173,650,753	2	91,621
B6xSPRET	Kidney	127,799,573	3	186,061
B6xSPRET	Liver	239,422,480	3	211,762
B6xSPRET	Lung	170,021,189	2	169,389

Supplementary Table 3. Summary of TF models.

Shown are TF motifs with enrichment of imbalanced SNVs. TF motifs were curated from multiple databases and annotated with gene name. Motifs with redundant sequence specificities by TOMTOM were identified and collapsed using a clustering approach[4].

		TFs overlapping sufficient variation		
	Total TFs in database	Human (166 individuals and 116 cell types[4])	Mouse (5 strains and 4 cell types)	Pooled human and mouse
TF motifs	2154	509	627	857
TF genes	695	268	335	430
TF motifs (collapsed)	270	82	105	131

Supplementary Table 4. Summary of mESC samples in this study.

Analyzed reads were required to pass all mapping filters. Read counts are in millions. Signal Portion of Tags (SPOT) scores are a measure of enrichment and refer to the proportion of reads mapping within DHS; SPOT scores are reported from hotspot V1.

Strain	Cell type	Experiment	Sample ID	# Sequenced reads (M)	# Analyzed reads (M)	# Duplicate reads (M)	SPOT
B6xCAST	mESC	DNase-seq	BS02277A	63	140	14	0.2798
B6xCAST	mESC	DNase-seq	BS02283A	192	128	11	0.2737
B6xCAST	mESC	CTCF ChIP-seq	BS03752A	61	39	24	0.6104

Supplementary Table 5. Summary of RNA-seq samples in this study.

Uniquely mapped reads were required to pass all mapping filters. Nonredundant reads exclude PCR duplicates. Read counts are in millions. Samples IDs beginning with SRR are from SRA study SRP020526[19].

Strain	Cell/tissue type	Sample ID	Num. pass filter alignments	Uniquely mapped reads	Nonredundant reads
B6xC3H	B cell	DS38895	199	173	94
B6xC3H	Kidney	DS38815	128	113	77
B6xC3H	Liver	DS38822	132	109	67
B6xC3H	Lung	DS38808	112	96	69
B6xCAST	B cell	DS35975	117	109	80
B6xCAST	Kidney	SRR823460	75	60	46
B6xCAST	Kidney	SRR823468	130	85	50
B6xCAST	Liver	SRR823469	213	148	88
B6xCAST	Liver	SRR823474	221	143	88
B6xCAST	Lung	SRR823447	86	74	55
B6xCAST	Lung	SRR823448	104	89	64
B6xPWK	B cell	DS36866	80	76	56
B6xPWK	B cell	DS37551	116	91	61
B6xPWK	Kidney	DS37491	112	107	74
B6xPWK	Liver	DS37504	124	100	55
B6xPWK	Lung	DS37484	100	92	73
B6xSPRET	B cell	DS39200	67	60	38
B6xSPRET	Kidney	DS37587	103	98	67
B6xSPRET	Kidney	DS38305	132	111	67
B6xSPRET	Liver	DS37600	94	87	54
B6xSPRET	Liver	DS38323	137	114	46
B6xSPRET	Lung	DS37580	125	115	70
B6xSPRET	Lung	DS38309	130	107	72

Supplementary Table 6. PCR primers.

Name	Sequence
P5	5’-AATGATACGGCGACCACCGAG-3’
P7	5’-CAAGCAGAAGACGGCATACGAG-3’
