# Supplementary material for: Tissue context determines the penetrance of regulatory DNA variation
Source: Nat Commun. 2021 May 14;12:2850. doi: 10.1038/s41467-021-23139-3 (PMC8121920; doi:10.1038/s41467-021-23139-3)
Supplement: Supplementary file 4 — Description of Additional Supplementary Files [file 41467_2021_23139_MOESM4_ESM.pdf]

Description of Additional Supplementary Files

File Name: Supplementary Data 1
Description: Details of sites tested for imbalance. Details of 357,303 SNVs tested for imbalance, including coordinates (mm10), read counts, P-value, and aggregate and per-cell/tissue type imbalance calls.
